# High Stakes Require More Than Just Talk: What to Do About Corruption in Health Systems: Comment on "We Need to Talk About Corruption in Health Systems"

**DOI:** 10.15171/ijhpm.2019.33

**Published:** 2019-06-02

**Authors:** Taryn Vian

**Affiliations:** School of Nursing and Health Professions, University of San Francisco, San Francisco, CA, USA.

**Keywords:** Risk Assessment, Anti-Corruption, Accountability, Transparency

## Abstract

Reluctance to talk about corruption is an important barrier to action. Yet the stakes of not addressing corruption in the health sector are higher than ever. Corruption includes wrongdoing by individuals, but it is also a problem of weak institutions captured by political interests, and underfunded, unreliable administrative systems and healthcare delivery models. We urgently need to focus on corruption as a health systems problem. In addition to supporting research to better understand the context and implications of corruption in health systems, this article suggests actions that public health professionals can do now to fight corruption.

Reluctance to talk about corruption is not new. Hutchinson et al identify five reasons why health policy-makers evade this pervasive problem.^1^ They highlight that corruption is difficult to define, and that what is called ‘corruption’ may really reflect coherent — even justifiable — actions to cope within severely dysfunctional systems. They observe that in documenting corruption problems, researchers must sort through incompatible and self-serving explanations, and may end up collaborating with corrupt officials to retain the access needed to work. Development partners may feel that bringing up such a sensitive topic might offend or damage relationships important to the success of projects. Questions about the legitimacy of studying corruption may be another reason people are reluctant to discuss the topic. Critics have suggested that anti-corruption dialogue is motivated by a neoliberal agenda to promote certain forms of social, political, and economic organization, not allowing for truly country-led development.^2,3^ Finally, our reticence reflects our chagrin at having very few evidence-based practices we can point to as solutions.

Robert Klitgaard wrote that the topic of corruption provokes evasion, excuses, and surprisingly little analysis.^4^ Excuses are plentiful: “corruption has existed forever,” “corruption greases the wheels of development.”^5^ Debunking excuses is tiresome, yet the stakes of not addressing health sector corruption are higher than ever. Corrupt countries have higher rates of infant, child, and maternal mortality even after adjusting for health care spending and other factors.^6,7^ Corruption in the health sector has been linked to higher rates of cancer death,^8^ antimicrobial resistance,^9^ anxiety,^10^ and lower patient satisfaction with and trust in the healthcare system.^11^ In Ukraine, efforts to fight corruption in procurement systems allowed the Ministry of Health to purchase more life-saving drugs and medical devices, reducing mortality for heart attack patients by 20%.^12^ Fighting corruption through anti-corruption measures such as fraud control and inspection, and indirectly through health systems strengthening, is a goal we must adopt now to save lives. Research is needed to determine the most efficient, effective, and feasible approaches in specific country contexts.

Though corruption can be tracked to individual leaders who put their personal interests above public duty, it is also a problem of weak institutions captured by political interests, and underfunded, unreliable administrative systems and healthcare delivery models.^13^ We urgently need to focus on corruption as a health systems problem. Yet increasing the pressure for accountability without dealing with inadequate financing can create perverse incentives. The US Veterans Administration is a cautionary tale: unrealistic performance goals and inadequate funding to reach those goals led employees to falsify data and hide performance failures.^14^

Anti-corruption strategies need to be informed by evidence, experience, and context. There is no magic bullet or one “right way” to fight corruption in the health sector. Hutchinson et al argue for convening key stakeholders to assess risks and reach agreement on the nature of the problem, followed by a priority-setting exercise to determine feasible remedies.^1^ Klitgaard also recommends participatory diagnosis of problems, noting that politicians and officials can be “remarkably forthcoming” in workshops, sharing detailed insights on where corruption can be found, why it exists, and what can be done to prevent it.^5^ Work in Albania found that citizens were not afraid to share experiences paying under-the-table for care that should have been free.^15^ Other research has suggested methods to reduce reticence of medical professionals in sharing information about accepting gifts and informal payments.^16,17^

Hutchinson et al recommend that researchers and policy-makers adopt a multi-disciplinary perspective when gathering and evaluating evidence of anti-corruption strategies, remarking that many pathways for research exist.^1^ While this is true, the time is ripe for convergence on methodologies to measure some problems, such as absenteeism, informal payments, and insurance fraud. Considering informal payments, for example, few studies have tried to separate the burdens posed by different types of payments such as cash payments, “bought and brought” supplies, and gifts.^18^ Rates may also vary due to methodological differences in recall periods, or the setting in which a payment was made (inpatient versus ambulatory care). Developing common indicators and survey research methodologies to allow cross-national comparison of the scope and nature of this and other problems will help us to evaluate whether health reforms are having the desired impact.

In 2015, former US President Barak Obama said, “*We’re on this planet a pretty short time, so that we cannot remake the world entirely during this little stretch that we have. But I think our decisions matter….[A]t the end of the day, we’re part of a long-running story. We just try to get our paragraph right.*”^19^ President Obama was aware that we inherit a world with “grudges, rivalries, hatreds, and sins of the past,” and there are limits to what we can fix and make better. Yet, there are things we can do, and this applies to seemingly intractable problems like corruption. We are part of a longer story, our decisions matter, and we need to work on getting our paragraph right. So, here are five things that public health professionals can do now to fight corruption.

1. Adopt an ethical frame when making decisions. Behavioral economists and business ethicists have studied how social norms and informal values exert a strong influence on people’s behavior, and how we frame problems influences how people think.^20^ Health leaders should stop to consider whether ethical principles are at stake when making decisions, especially in designing changes to administrative systems. Small signals, such as where and when we sign a form, can have an impact in promoting integrity.^21^

2. Assess risks of corruption. Health professionals who are part of a team starting a new project or program should ask about whether the staff are getting adequate training to assure that staff interests are aligned with the project’s goals. It is important to ask about oversight procedures, and checks and balances to assure that people are able to resist temptation to engage in wrongdoing. Minimizing occupational temptation helps everyone.^22^

3. Strengthen accountability in programs. Health professionals should understand the accountability structures in their programs. Who is accountable to whom, for what, and ‘so what’ (eg, what are the consequences for failures in accountability)? Start a discussion of ways to create fairer processes and to reward good performance or identify and address performance that is not up to standard. Suggest activities that will bring the people in power into closer touch with those people who depend on their decisions (lower level staff, constituencies).^23^

4. Value and model transparency. Health professionals at all levels of an organization can encourage active internal and external transparency and information sharing. According to the Association of Certified Fraud Examiners, the most effective measures for fraud control include proactive data monitoring and analysis, management review of accounts and transactions, and hotlines (each producing 50%-54% reduction in median losses).^24^ For a team leader, this may just involve telling your team members what to do if they are not paid on time, or they see something that seems unfair or not right. Do not assume people know how to complain. In programs with formal complaint hotlines, it is important to test the hotline periodically to be sure it is working. Health research should experiment with methods to adapt complaint mechanisms successfully to different local contexts.

5. Support and involve civil society organizations in your work. Health professionals should encourage partnerships with community-based organizations that are trying to empower citizens, whether they currently work in the health sector or not. The health sector is complicated, and corrupt actors take advantage of this complexity to hide their malfeasance. Help anti-corruption non-governmental organizations to become more knowledgeable about health issues. The more citizens, community-based organizations, and non-governmental organizations know about how the sector is organized and financed, the more easily they can identify and call attention to corruption risks.

While it may still be uncomfortable to talk about corruption, we must have these difficult conversations and move beyond them to action. We need to foster opportunities to discuss problems together, to build on-the-ground knowledge. Corruption is a social determinant of health. It is a cost that rarely is quantified in our cost-effectiveness analyses, a risk often is not mentioned in project appraisal reports. Yet, corruption is a real barrier preventing us from achieving our goals in public health. We need to work harder to understand why corruption happens, and to take actions — as health and development professionals, as government officials, and as citizens — to prevent it.

## Ethical issues

Not applicable.

## Competing interests

Author declares that she has no competing interests.

## Author’s contribution

TV is the single author of the paper.
